# Why coaching matters: exploring the interplay of teacher self-regulation and well-being with a longitudinal multigroup model

**DOI:** 10.3389/fpsyg.2025.1647838

**Published:** 2025-09-26

**Authors:** Zippora Bührer, Christine Wolfgramm, Simone Berweger, Andrea Keck Frei, Christine Bieri Buschor

**Affiliations:** Zurich University of Teacher Education, Zurich, Switzerland

**Keywords:** teachers’ self-regulation, emotional exhaustion, work engagement, well-being, coaching, multigroup SEM

## Abstract

**Introduction:**

Self-regulation is considered an important aspect of professional competence that promotes teachers’ well-being. It involves controlling thoughts, feelings and actions to pursue goals, deal adaptively with challenges and cope with stress. For early career teachers, these skills are crucial for their health and staying in the profession. However, longitudinal studies which position self-regulation as a personal resource for teachers’ well-being remain scarce. The aim of our study was to examine the reciprocal interplay between teachers’ self-regulation and well-being (i.e., emotional exhaustion and work engagement), and the impact of self-management training and subsequent professional online coaching on these relations.

**Methods:**

The study was conducted as part of a professional development course for early career teachers (*N* = 273), in which the participants were randomly assigned to a standardized training program. Using multigroup structural equation modeling, we compared two treatment groups (training-only, training plus online coaching) with a control group regarding the structural relations.

**Results:**

The model comparison revealed significant differences: Self-regulation predicted both work engagement and emotional exhaustion, but only in the group that received training plus coaching. Furthermore, work engagement predicted self-regulation across all groups.

**Discussion:**

We conclude that self-regulation can serve as an effective personal resource for teachers well-being, under the condition that it is activated as resource and supported. In pursuing challenging goals, coaching may offer crucial support in each phase of the self-regulation process. This longitudinal study contributes to a differentiated view of self-regulation in the field of professional development, and clarifies the conditions under which it serves as an effective individual resource for teachers well-being.

## 1 Introduction

Subjective well-being can be seen as a central goal in itself (Diener, 2000; Seligman and Csikszentmihalyi, 2000), but in relation to teachers, well-being is also linked in many ways to professional performance and various aspects of teaching quality. Emotionally exhausted teachers are less able to adapt the pace of teaching to the needs of their students (Klusmann et al., 2008), to provide emotional support and to organize lessons, which in turn is linked to student performance (Klusmann et al., 2009). Teachers’ well-being is also related to the intention to remain in the profession (Skaalvik and Skaalvik, 2016, 2018). Teachers professional well-being and its improvement have recently received increasing attention, as research findings indicate that teachers face high work pressure, lack of recovery time and exhaustion (Sandmeier et al., 2020) and overall experience higher levels of stress than other occupational groups (Iriarte Redín and Erro-Garcés, 2020). High levels of stress, when not successfully managed, increase the risk of reduced work engagement and emotional exhaustion (Hobfoll, 1989). This underscores the importance of specific actions to improve teachers’ well-being.

The teaching profession involves a variety of complex demands (García-Carmona et al., 2019) that can be highly stressful (e.g., Viac and Fraser, 2020), especially for beginning teachers (Dicke et al., 2018; Klusmann, 2011). Risk factors for teacher stress and exhaution include discipline problems and disruptive attitudes and behavior by students, heterogeneity in the classroom and differences in student motivation and performance, time pressure, and dealing with continual education reforms as well as conflicts with superiors, co-workers and parents (Berweger et al., 2019; García-Carmona et al., 2019). The multidimensional concept of teachers’ well-being is frequently operationalized as the absence of persistent negative affect, such as emotional exhaustion and burnout. However, research has increasingly shifted toward positive expressions of well-being, such as work engagement. According to the Job Demands-Resources model (JD-R), work-related well-being depends on job characteristics – specifically job demands and job resources (Bakker and Demerouti, 2007) – but also on personal resources (Xanthopoulou et al., 2007). One important personal resource for coping with these high demands and maintaining teacher well-being is self-regulation (Klusmann et al., 2009; Mattern and Bauer, 2014; Philipp and Kunter, 2013). Self-regulation can be defined as an aspect of teachers’ professional competence that enables them to control and monitor their own thoughts, emotions, and behavior in the pursuit of short- or long-term goals (Zimmerman, 2000).

Professional development training programs, including coaching, have been established worldwide to support self-management and well-being, and to retain teachers in the profession by preventing burnout (Darling-Hammond et al., 2017). It has been shown that (student) teachers benefit from self-regulation training (Celebi et al., 2014; Mattern, 2012; Li Sanchez and Schwinger, 2024), and that coaching can be an effective support for teacher professional development (Kraft et al., 2018). However, there is little empirical evidence that training programs that strengthen self-regulation and support its transfer through coaching can improve in-service teachers’ well-being.

In this longitudinal study, we analyze the complex interplay of self-regulation and teacher well-being in terms of work engagement and emotional exhaustion and how self-management training, including subsequent coaching, affects this interplay.

### 1.1 Teacher well-being

Teacher well-being is often understood primarily as an affective state, measured as the absence of perceived stress and emotional exhaustion (Klusmann et al., 2008). However, there are broader conceptualizations of teacher well-being that also include motivational and behavioral aspects (Fox et al., 2023; Hascher and Waber, 2021). Bakker and Oerlemans (2011) developed a model of occupational well-being focusing on work engagement, characterized by high levels of positive emotions and energy during work. This understanding of work-related well-being is based on the JD-R model, which proposes a health-promoting and a health-damaging process as relatively independent of each other (Bakker and Demerouti, 2007; Bakker and Demerouti, 2017). Accordingly, job resources and personal resources initiate a gain cycle that is linked to high levels of work engagement and job satisfaction, whereas stressful working conditions lead to a vicious circle of emotional exhaustion and burnout (e.g., Bakker and Demerouti, 2007, 2017). Current versions of the JD-R model also propose reciprocal effects: Exhaustion is associated with reduced self-regulation (self-diminishing), while work engagement favors better self-regulation (proactive behavior) (Bakker and Demerouti, 2017; Bakker et al., 2023a). Findings have confirmed that people with an increased job strain are less likely to use adaptive self-regulation strategies (Bakker and De Vries, 2021; Bakker et al., 2023b).

Emotional exhaustion describes a negative psychological state that occurs when job demands exceed available resources (Hobfoll, 1989) over a longer time. Studies indicate that emotional exhaustion in teachers is closely tied to their working conditions (Pogere et al., 2019; Skaalvik and Skaalvik, 2015) but can also be positively influenced by personal resources like self-regulation (Mattern and Bauer, 2014). In addition, highly exhausted teachers appear to have lower work engagement (Skaalvik and Skaalvik, 2016).

Schaufeli et al. (2002) defined work engagement as “a positive, fulfilling, work-related state of mind that is characterized by vigor, dedication, and absorption” (p. 74). Vigor is considered the direct opposite of emotional exhaustion, which represents the core dimension of burnout (Schaufeli et al., 2006). Teachers with high work engagement feel full of energy, work passionately and are absorbed in their work. In addition, they have great perseverance in overcoming difficulties (Greenier et al., 2021). Work engagement can be predicted by job resources, as well as personal resources (Mazzetti et al., 2023). Simultaneously, high engagement contributes to “job crafting,” whereby individuals with high engagement manage to create a resourceful and challenging work environment for themselves by improving their resources, such as self-efficacy, while reducing obstructive work demands (Vogt et al., 2016). Findings show the importance of personal resources such as self-efficacy and optimism for work engagement (Bakker and Albrecht, 2018; Bakker and Sanz-Vergel, 2013; Bakker and van Wingerden, 2021; Mazzetti et al., 2023; Xanthopoulou et al., 2009). However, limited attention has been paid to the specific impact of self-regulation.

### 1.2 Teachers’ self-regulation

In the context of teacher education, teachers’ self-regulation has primarily been discussed with reference to Zimmerman’s (2000) concept (Kunter et al., 2013) based on Bandura’s (1986) approach of identification with goals. Zimmerman (2000) describes self-regulation as a cyclical process with the three phases of (a) goal setting and planning, (b) performing the action while simultaneously monitoring and controlling attention and volition, and (c) self-reflection. These phases are linked via feedback loops and each requires specific strategies involving cognitive, metacognitive, affective and behavioral regulation, including the ability to cope with challenges and successfully manage stress (Kunter et al., 2013) to remain healthy. Whereas this approach focuses on persistence, some goal theories also include disengagement. From this perspective, goal striving unfolds in a continuous process of persistence and disengagement; individuals commit to goals, invest and withdraw resources, increase and reduce efforts and let go of goals or change them. Persistence and disengagement are both functional and represent important aspects of self-regulation (Brandstätter and Bernecker, 2022). Motivational theories, such as personality systems interaction (PSI) theory (Kuhl, 2000) and the model of action phases (MAP) (Gollwitzer, 2012) that are rooted in common origins (Brandstätter and Bernecker, 2022), adress these processes and focus on volition. PSI theory includes the following dimensions of self-regulation: (a) selecting self-congruent goals, (b) pursuing a goal over a long period of time and (c) not giving in to one’s immediate impulses or when coping with difficulties (Kuhl, 2000). MAP theory describes the process from wishes to action, including four phases: deliberation, planning, action and evaluation. The theory was extended by combining two self-regulation strategies (MCII): mental contrasting (Oettingen, 2015) and implementation intentions (Gollwitzer, 2012), which are described below. These strategies can be applied to deal with the challenges of (a) committing oneself to desirable goals, (b) initiating goal-directed action, (c) remaining persisting while facing difficulties and even (d) stopping unsuccessful goal pursuit.

Teachers’ self-regulation has been described as an active process in which teachers use adaptive regulation strategies to achieve professional goals and deal with obstacles (Capa-Aydin et al., 2009; Mattern, 2012; Mattern and Bauer, 2014). Self-regulation is particularly important in the context of the teaching profession, where individuals are confronted with challenging professional demands and conflicting goals (Sandmeier et al., 2020). Teachers’ self-regulation has been found to be related to lower emotional exhaustion in cross-sectional studies (Klusmann et al., 2008; Kunter et al., 2013; Mattern and Bauer, 2014). There is still a lack of research on the role of self-regulation abilities in relation to teachers’ work engagement. A study by De Stasio et al. (2019) found that self-regulation combined with co-regulation, in terms of peer support, was cross-sectionally related to pre-school teachers’ work engagement. On this basis, we assume that self-regulation acts as a personal resource that reduces teachers’ emotional exhaustion and, at the same time, has a positive effect on their work engagement.

### 1.3 Strengthening self-regulation through training and coaching

Self-regulation in the process of goal pursuit can be fostered through training in strategies that enable (1) realistic goal setting, (2) goal commitment and ongoing engagement for goal-directed actions, and (3) monitoring one’s own goal attainment as well as goal adjustment (Gollwitzer, 1990; Oertig et al., 2013). Training in such strategies and methods that facilitate appropriate goal setting and activate goal-related resources can improve well-being and change the experience of stress when overcoming challenges in the pursuit of goals (Ehrlich, 2023; Sheldon et al., 2002). Methods that have proven to be particularly effective in strengthening self-regulation are if-then plans (Achtziger et al., 2008; Thurmer et al., 2013). They are based on mentally contrasting the imagined positive effect after goal achievement compared to the current state (Oettingen, 2015; Wieber et al., 2014). Training to strengthen self-regulation has proven effective for (student) teachers in terms of improving self-regulation (e.g., Celebi et al., 2014; Mattern, 2012), reducing occupational stress and improving well-being (Li Sanchez and Schwinger, 2024). A meta-analysis on the effectiveness of interventions to reduce teacher burnout found that cognitive-behavioral interventions involving cognitive restructuring, goal setting and planning, and problem-solving training had a significant impact on emotional exhaustion (Iancu et al., 2018). However, intervention studies supporting early career teachers’ self-regulation in goal-oriented management of work-related challenges remain scarce. In particular, the role of coaching to support the development of self-regulation remains understudied (Barato and Rodríguez Moneo, 2022).

Coaching, understood as an individually tailored intervention strategy, seems promising for ensuring the transfer of skills acquired in training into practice (Klusmann et al., 2008; Rzejak et al., 2013). Professional coaching supports the setting, pursuit and achievement of goals in coping with challenges (Grant, 2014), which improves well-being, helps manage work demands (Van Zyl et al., 2020) and promotes the experience of competence. Coaching is understood as a collaborative relationship between a client and coach, characterized by (1) the maintenance of cognitive and emotional support, (2) setting and pursuing personal goals and (3) co-creative problem-solving to strengthen resources through a development process that involves the provision of skills, methods and reflection (Greif et al., 2022; Van Zyl et al., 2020). Recent studies emphasize the interplay between coaches’ provision of support and coachees’ self-regulation. Caregivers in management positions who received five coaching sessions, for example, showed improved self-management competencies, including self-regulation. The latter contributed to the prediction of goal attainment (Mühlberger et al., 2024). Fingas et al. (2025) found that coaches’ support and open questions elicited female coachees’ self-regulation. The competence of the coach, along with a combination of different methods, is decisive for the effectiveness of coaching (Jones et al., 2016; Grant, 2014). Furthermore, the relationship between coach and coachee, as well as the voluntary nature of the coaching influence its success, whereas the duration of the coaching has proven to be insignificant (Grant, 2012; Greif et al., 2022; Jones et al., 2016). Online coaching represents a more flexible form of coaching, offering low-threshold interventions to support teachers’ professional development (Barrett et al., 2024; Crawford et al., 2021; Powell and Bodur, 2019). It seems to be as effective as face-to face coaching in terms of satisfaction and goal achievement (Atad and Grant, 2021; Jones et al., 2016; Greif et al., 2022). Furthermore, online coaching supports self-regulation strategies, self-reflection, and their transfer to everyday working life (Jones et al., 2016; Richards and Viganó, 2013).

### 1.4 Self-management training for early career teachers

In the context of a larger research project, a self-management training program (SMT) was developed at the Zurich University of Teacher Education to support early career teachers in dealing with personally demanding challenges (Keck Frei et al., 2020). The SMT is based on existing goal-oriented self-management training programs for professionals (Kanfer et al., 2006) and includes elements from effective self-regulation training for teachers in the German-speaking context (Schaarschmidt and Fischer, 2013; Celebi et al., 2014; Mattern, 2012). Additionally, the SMT incorporated the self-regulation strategy of mental contrasting (Oettingen et al., 2015), combined with implementation intentions (MCII) (Oettingen and Gollwitzer, 2001), and if-then plans (Achtziger et al., 2008; Thurmer et al., 2013). Furthermore, (peer) coaching methods to strengthen teachers’ resources and goal orientation from the field of positive psychology (Richter et al., 2021) were included. The training was complemented with subsequent professional online coaching to facilitate the transfer to everyday work.

The SMT consisted of three consecutive modules (three half-days; for the training manual and workbook see Bieri Buschor et al., 2022a, 2022b). First, participants analyzed their work-related behavior and learning patterns, with a focus on activating their own resources. Second, they learned about different self-regulation strategies, from metacognitive to emotion regulation strategies, and how to apply them to challenging work situations. This module included exercises to support the development of strengths, self-reflection and goal setting. Third, they set personal goals for their professional behavior (e.g., applying strategies to improve health-oriented behavior) defined sub-goals and developed an action plan, where they anticipated obstacles to goal achievement as well as strategies to deal with them. From a process perspective, module three guided the setting of goals, strengthened goal commitment and prepared participants for coping with challenges regarding the implementation of the action plan. To enhance the long-term sustainability of training and coaching effects, participants were supported to use peer-coaching methods to carefully deliberate, plan and cope with anticipated challenges. The aim was also to strengthen their public commitment, thereby supporting their persistence and enabling them to continue implementing professional development goals into practice in the future.

The online coaching is based on a goal-oriented relationship to support transfer learning (Greif et al., 2022) and thus the next phase of the self-regulation process that has been initiated in module three. The focus of the coaching was on goal implementation, support in the pursuit of goals and their adaptive adjustment. Additionally, coaches could address further questions and support coachees with specific coaching methods (e.g., inner team, system visualizations). Lecturers with a professional systemic coaching background and long-term experience led the SMT groups and served as coaches. The online coaching took place via the coaching software cai-world (www.cai-world.com), which provides professional coaching methods and reflection tools (Berninger-Schaefer, 2018).

### 1.5 The present study

Based on the explanations and empirical findings outlined above, the present study has two main aims. First, it aims to better understand the longitudinal interplay between self-regulation, work engagement and emotional exhaustion. The focus is on the role of self-regulation for emotional exhaustion and engagement as it is assumed to be an important personal resource in maintaining teacher well-being. Second, it aims to gain insight into the effects of training plus coaching on these relations.

Accordingly, the research questions are as follows:

*RQ1:* How do teachers’ self-regulation, emotional exhaustion and work engagement interact over time?

*RQ2:* What is the impact of the SMT and subsequent online coaching on the structural relationships between self-regulation, emotional exhaustion and work engagement?

**Figure 1:**
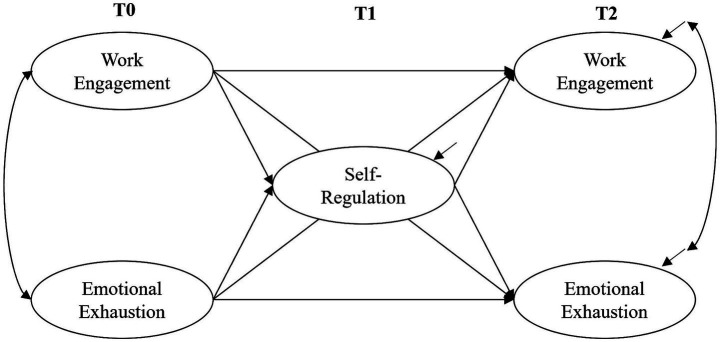
Longitudinal model of self-regulation, work engagement and emotional exhaustion, tested across time (T0, pretest; T1, posttest 1 after SMT; T2, posttest 2 after 5 months’ online coaching) and group (CG, control group; TG, training group; TCG, training plus coaching group).

To address these research questions, the study tests a model of the interplay between the three constructs over time for multiple treatment groups (Figure 1). Based on the research of Mattern and Bauer (2014), which showed positive effects in a cross-sectional study, we expected teachers with higher self-regulation at T1 to be less emotionally exhausted at T2.

The meta-analysis of Mazzetti et al. (2023) highlights the significance of personal resources (resilience, self-efficacy, optimism, proactivity) for work engagement. As self-regulation is another important personal resource, and there are initial indications of corresponding effects on work engagement (De Stasio et al., 2019), we expected that teachers with higher self-regulation at T1 would tend to be more engaged in their work at T2. Furthermore, in line with the theoretical assumptions of the JD-R framework (Bakker and Demerouti, 2017), we also analyzed the reciprocal relationships between emotional exhaustion, work engagement, and self-regulation. The longitudinal design with three measurement points also allowed us to test indirect effects and thus whether self-regulation may play a mediating role. This is supported by theoretical assumptions (e.g., Cramer et al., 2018) that (personal) resources have this mediating function on emotional exhaustion and potentially also on work engagement.

We consider self-regulation as a dynamic adaptive process that stresses motivational and volitional aspects. In agreement with van Zyl et al. (2020), we assumed that action- and process-oriented training, especially in combination with coaching, can enhance well-being by strengthening self-regulation strategies and making their use more likely and effective. Accordingly, we expected that participating in the SMT with and without coaching would strengthen the positive relation between teachers’ self-regulation and work engagement and the negative relation between self-regulation and emotional exhaustion.

In summarizing our research hypotheses, we expected teachers with higher self-regulation at T1 to be less emotionally exhausted at T2 (*hypothesis 1*) and having a higher work engagement at T2 (*hypothesis 2*). Furthermore, we hypothesized that participation in treatment with SMT (TG) alone and with SMT in combination with coaching (TCG) would strengthen the positive relationship between teachers’ self-regulation and their work engagement (*hypothesis* 3a), as well as the negative relationship between self-regulation and emotional exhaustion (*hypothesis* 3b).

## 2 Materials and methods

The present study is part of a research project funded by the Swiss National Science Foundation (2018–2022). The described self-management training program (SMT, see Section 1.4) was embedded in a three-week professional development (PD) program for early career teachers at the Zurich University of Teacher Education (Keck Frei et al., 2020) and it comprises content-focused courses, workshops, and collaborative projects. The online coaching started after the PD program and was offered over a period of five months (180 min over 3–6 sessions). Participants completed online questionnaires at three time points: the first (T0) in November 2017 at a pre-course information session for the PD program, the second (T1) in January 2018 on the last day of the program, and the third (T2) in June 2018 after the five-month implementation and online coaching phase.

### 2.1 Participants

The sample consisted of *N* = 273 kindergarten and primary school teachers (95% female, grades 1 to 6, pupils aged 4–12 years) from the canton of Zurich (Switzerland), who participated voluntarily in the three-week PD program and agreed to participate in the study (informed consent). They completed their teaching diploma at the primary or kindergarten level (73 and 27% respectively; including 10% career changers) between 2012 and 2016, with the majority in 2015. At the time of the survey, teachers were on average 28 years old (*SD* = 6.18; range: 23–53 years). They worked on average 87% of a full-time equivalent position (*SD* = 12.89). The participants were randomly assigned to the three conditions of the study: The training group (TG, *N* = 95) participated in the SMT, the training plus coaching group (TCG, *N* = 60) received online coaching in addition to the SMT during the implementation phase, and the control group (CG, *N* = 118) participated in the PD program only. Among those in the TCG 68% participated in three coaching sessions, 7% attended four to five sessions, 13% in two sessions and 12% in one. Of the initial *N* = 79 teachers assigned to the online coaching, *N* = 19 did not participate for various reasons (e.g., termination or interruption of employment, time constraints and no interest in coaching). Dropout analyses revealed no differences between the teachers who completed the online coaching and those who quit with regard to the effects of demographic variables (gender, age, school level, employment) and the three latent variables at baseline measurement (emotional exhaustion and work engagement at T0, self-regulation at T1).

### 2.2 Measures

*Self-regulation* was measured with the instrument of Mattern and Bauer (2014), a validated questionnaire on the cognitive aspects of teachers’ self-regulation, which is based on Kuhl and Fuhrmann (1998) Volitional Components Inventory and items from Schwarzer’s Self-regulation Scale (1999). The combination of exploratory factor analysis (EFA) and confirmatory factor analysis (CFA) confirmed the factor structure and the subscales except for two items. These items were replaced with items from the original Version of Schwarzer (1999), resulting in good overall internal validity of the scale. The final scale consisted of eight items distributed across three subscales, representing three aspects of self-regulation: *action planning* (e.g., “Before I start an extensive task, I determine how I will proceed”), *self-motivation* (e.g., “In a difficult activity, I can specifically look at the positive sides”), and *attention control* (e.g., “I can keep my mind from constantly wandering from the task at hand”). Response scales ranged from 1 (*disagree*) to 4 (*agree*). Reliability (Cronbach’s *α*) was *α* = 0.791.

*Work engagement* was assessed using two sub-scales from the German Version of the Utrecht Work Engagement Scale (UWES-9, Schaufeli et al., 2006): *vigor* (e.g., “At my work, I feel bursting with energy.”) and *absorption* (e.g., “I am immersed in my work.”). Three items from each subscale were used and rated on a scale from 1 (*does not apply*) to 4 (*applies*). Reliability was *α* = 0.770 at T0 and *α* = 0.815 at T2.

*Emotional exhaustion* was measured using a combination of items from the German version of the Maslach Burnout Inventory (MBI; Baumert et al., 2008) and the job stress inventory of Enzmann and Kleiber (1989). Two of the MBI items had to be excluded due to skewed distribution and lack of content validity. We supplemented the scale with suitable items from Enzmann and Kleiber. Exploratory factor analysis (EFA) and confirmatory factor analysis (CFA) confirmed a one-dimensional scale at both test points. This enabled us to form three parcels as indicators in the next step (see Section 3.3). The five items from Enzmann and Kleiber’s emotional exhaustion scale (1989) – e.g., “I often feel overwhelmed.” – were rated from 1 (*does not apply*) to 5 (*applies*). The two items from Baumert et al. (2008) – e.g., “I often felt exhausted at school.” – were rated from 1 (*does not apply*) to 4 (*applies*). Due to the different metrics of the scales, all items were z-transformed. Reliability was *α* = 0.845 at T0 and *α* = 0.873 at T2 (for detailed information on all measures see the Supplementary material).

### 2.3 Data analyses

To analyze the longitudinal interplay of the constructs (Figure 1, *RQ1*), we tested a multigroup cross-lagged panel model with three latent variables: teachers’ *self-regulation*, *work engagement* and *emotional exhaustion*. The model was tested across the three groups (CG, TG, TCG) and specified so that the values of two latent variables – *work engagement* and *emotional exhaustion* – predicted their own subsequent values, thus measuring the stability from T0 to T2. Cross-lagged effects were estimated between all three latent variables. Accordingly, each cross-lagged effect on *work engagement* and *emotional exhaustion* at T2 was controlled for its corresponding baseline level at T0. To test for a possible mediating role, self-regulation was integrated into the model at T1.

This multiple group structural equation model (SEM) allowed us to examine the impact of the treatment conditions and it provided information on whether SMT alone or combined with online coaching influenced the development of work engagement and emotional exhaustion over time (*RQ2*). Descriptive analyses, reliability calculations and crosstabulations with subsequent χ^2^ tests were conducted using IBM SPSS 28. All other analyses were performed using Mplus 8 (Muthén and Muthen, 2017). We used robust maximum likelihood estimation (MLR) to correct for non-normally distributed data (Satorra and Bentler, 1994). Using full information maximum likelihood estimation (FIML) allowed cases with missing values to be included in the analyses (Schafer and Graham, 2002). The percentage of missing values for the full model was 3% at both T0 and T1. At T2, five months after SMT, 27% of all maximum possible data points were missing.

After conducting confirmatory factor analysis (CFA) for all variables and time points, we modeled the latent variables per construct individually. To estimate the complex longitudinal multigroup model with a total of five latent variables with a rather small sample (*N* = 273, smallest group size *N*_TCG_ = 60), the number of model parameters had to be reduced. To do this, we used item-parceling (Little et al., 2002) to build three indicators per latent construct. While the parcels for emotional exhaustion and work engagement were formed with a balancing approach, we chose a content-driven approach for self-regulation, with each parcel representing a subscale. In a next step, we modeled the complete model shown in Figure 1 with the full sample. As is common in longitudinal studies, the residuals of the same indicators (in our case, parcels) were correlated across time points in our model (Geiser, 2010).

To analyze the reciprocal associations between the three constructs in the multigroup model, we had to ensure that the factorial structure of the latent variables was sufficiently equivalent across time points (for work engagement and emotional exhaustion) and groups. We therefore modeled the multigroup SEM and tested whether strong longitudinal and multigroup measurement invariance was met simultaneously (see Section 3.4). In the next step, we examined the effects between the three constructs using effect coding (Little, 2013). Indirect effects were estimated using the bias-corrected bootstrap method with 5,000 bootstrap resamples (Geiser, 2010). We evaluated the goodness of fit for all models using the χ^2^ test, the comparative fit index (CFI) and the standardized root mean square error of approximation (RMSEA). As the χ^2^ test is known to be sensitive to sample size, comparative and absolute goodness of fit indices were used in addition to check model fit (West et al., 2023). According to Little (2013), in the context of longitudinal and multigroup models, CFI values above 0.90 indicate an acceptable model fit while values of 0.95 or higher indicate a very good model fit. For the RMSEA, models with values between 0.08 and 0.05 suggest an acceptable model fit, and values of 0.05 or lower suggest a good model fit.

Satorra-Bentler’s scaled (mean-adjusted) χ^2^ difference test [p(∆χ^2^); Satorra and Bentler (2010)] was used to test the degrees of measurement invariance (configural, metric, scalar) across time and group (see Section 2.4) as well as to test the equality of significant paths in the regression model between groups (see section 3.2). Accordingly, changes in model fit were evaluated by comparing the less restrictive model with the more restrictive one. As long as the *∆*χ^2^ does not indicate a significant decrease in fit, a higher level of invariance can be assumed (Byrne, 2012). When testing for group differences, a significant ∆χ^2^ result after setting equality constraints for the paths to be tested between groups confirms a significant difference.

For the interpretation of the standardized regression coefficients, Keith (2015) recommends classifying *β* < 0.10 as a small effect, *β* between 0.10 and 0.25 as a moderate effect and *β* > 0.25 as a strong effect. For longitudinal cross-lagged effects, we follow Orth et al. (2024), who recommend interpreting standardized regression coefficients of *β* = 0.03 as small, *β* = 0.07 as medium, and *β* = 0.12 as large effects.

### 2.4 Measurement invariance over time and group

Following Little (2013) we first specified the multigroup model and tested for configural invariance with all variables in the model (factor loadings and intercepts of indicators are freely estimated across the two time points and for each group; latent means were fixed to 0, latent variances to 1). As a second step, we tested metric invariance by equating the factor loadings of the corresponding indicators across the two time points and all groups. Latent means were fixed at 0 and latent variances were freely estimated, except for the first group, where for each latent construct the variance at the first time point (T0) was fixed at 1. As a third step, we tested scalar invariance by equating both factor loadings and intercepts of corresponding indicators across time points and groups. In addition, latent means and variances were freely estimated, except for the first group, where at the first time point (T0) they were fixed at 0 and 1, respectively (Little, 2013).

The fit of the configural, metric and scalar invariance models was acceptable, and the changes in fit in the metric and scalar invariance models as determined by *∆*χ^2^ were not significant (Table 1). Thus, scalar measurement invariance over time and group was supported. This allows for the comparison of the cross-lagged panel model and the relations among work engagement, emotional exhaustion and self-regulation across the three groups (CG, TG, TCG).

**Table 1:** Test of measurement invariance for the full model across time (T0 – T1 – T2) and group (control, training, training plus coaching).

Model	χ^2^	df	p	Corr. MLR	CFI	RMSEA (90% CI)	Cfit	p(∆χ^2^)
Configural invariance	341.574	222	0.000	0.942	0.927	0.077 (0.060–0.093)	0.005	
Metric invariance	364.817	246	0.000	0.967	0.927	0.073 (0.057–0.088)	0.013	0.360
Scalar invariance	370.076	270	0.000	0.970	0.939	0.064 (0.047–0.079)	0.087	0.999

*N* = 273; MLR, maximum likelihood estimation with robust standard errors; df, degrees of freedom; Corr. MLR, H1 scaling correction factor for MLR; CFI, comparative fit index; RMSEA (90% CI), root mean square error of approximation and its 90% confidence interval; CFit, test of close fit (likelihood that population RMSEA <0.05); p(∆χ^2^), Satorra-Bentler scaled χ^2^ difference test.

## 3 Results

### 3.1 Preliminary analyses

The intercorrelations between the latent variables self-regulation at T1, work engagement at T0 and T2 and emotional exhaustion at T0 and T2 for the three groups (CG, TG, TCG) are presented in Table 2. Almost all variables showed moderate to strong correlations (Cohen, 1992) in the theoretically expected direction. The strongest values were observed for the same correlations over time: work engagement (*r* range: 0.591 to 0.916) and emotional exhaustion (*r* range: 0.587 to 0.835). Correlations between self-regulation and work engagement ranged from moderate to strong. Correlations between self-regulation and emotional exhaustion indicate differences between the groups, with *r* between −0.206 and −0.543. Correlations between work engagement and emotional exhaustion ranged from moderate to strong.

**Table 2:** Descriptive statistics and intercorrelations of all latent variables for three groups.

Variables	*M (SD)*	1	2	3	4
Control group (*N* = 118)					
1. Self-regulation T1	3.10 (0.34)	–			
2. Work engagement T0	3.17 (0.36)	0.653***	–		
3. Work engagement T2	3.12 (0.45)	0.640***	0.916***	–	
4. Emotional exhaustion T0	−0.02 (0.75)	−0.375**	−0.411***	−0.411***	–
5. Emotional exhaustion T2	0.05 (0.91)	−0.444***	−0.306**	−0.410***	0.835***
Training group (*N* = 95)					
1. Self-regulation T1	3.05 (0.27)	–			
2. Work engagement T0	3.00 (0.31)	0.415**	–		
3. Work engagement T2	3.05 (0.38)	0.467**	0.591**	–	
4. Emotional exhaustion T0	−0.01 (0.83)	−0.206	−0.294*	−0.343*	–
5. Emotional exhaustion T2	−0.03 (0.81)	−0.401*	−0.305*	−0.592***	0.735***
Training plus coaching group (*N* = 60)					
1. Self-regulation T1	3.00 (0.32)	–			
2. Work engagement T0	2.99 (0.40)	0.570***	–		
3. Work engagement T2	2.96 (0.33)	0.883***	0.833***	–	
4. Emotional exhaustion T0	−0.00 (0.84)	−0.311*	−0.482***	−0.242*	–
5. Emotional exhaustion T2	−0.01 (0.85)	−0.543**	−0.219^+^	−0.303*	0.587***

M, mean; SD, standard deviation; T0, pretest; T1, posttest 1 after SMT; T2, posttest 2, after 5 months online coaching; ^+^*p* < 0.10; *p < 0.05; **p < 0.01; ***p < 0.001 (two-tailed).

To analyze differences in demographic variables between the groups (randomization check), a series of cross-tabulations and χ^2^ tests were conducted. These tests revealed no differences in characteristics such as gender, age, school level, and employment. When testing for group differences in the three latent variables of the model in the baseline survey, it was found that participants in the CG showed a significantly higher mean for work engagement (T0, *M* = 3.17, *SD* = 0.36) than those in the TG (*M* = 3.00, *SD* = 0.31) and TCG (*M* = 2.99, *SD* = 0.40; see Table 2). There were no significant differences in mean values for emotional exhaustion and self-regulation (T1).

### 3.2 Main analyses

**Figure 2:**
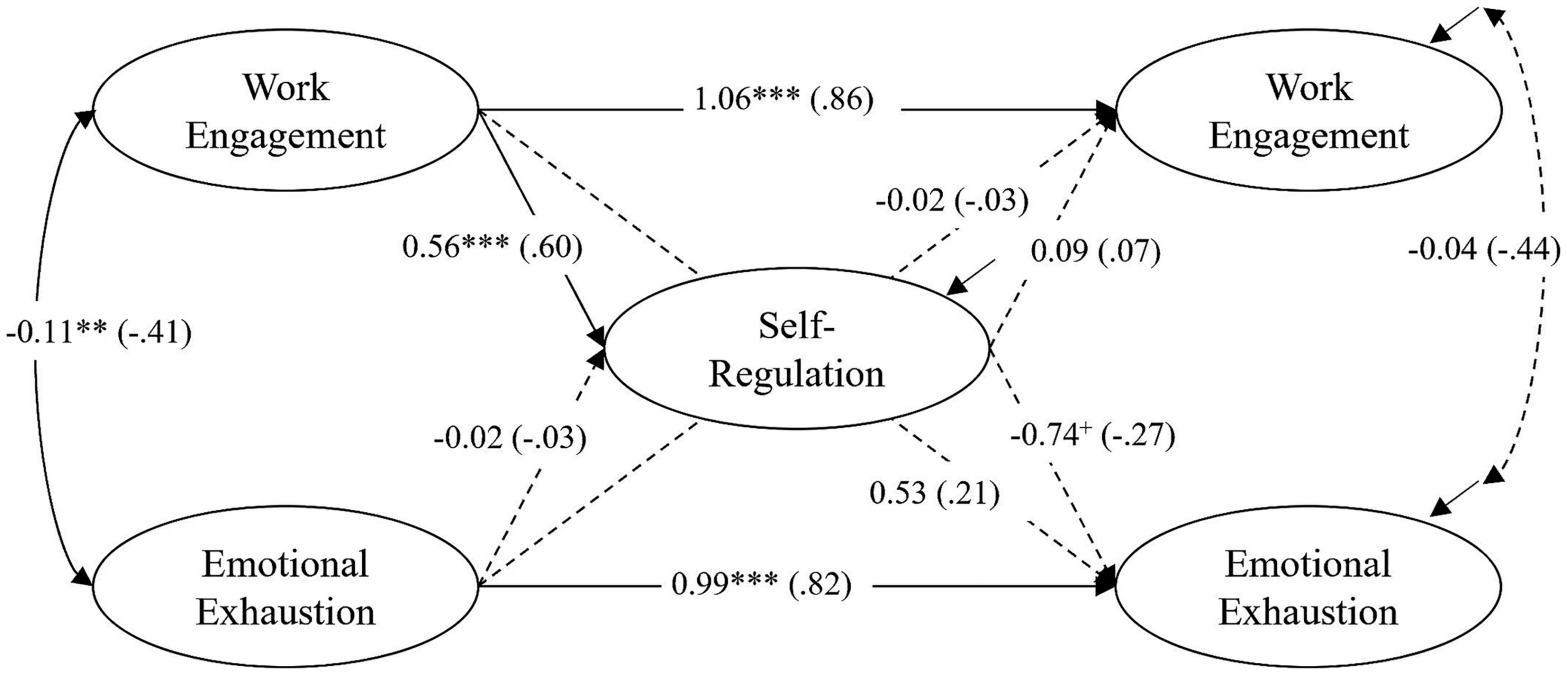
CG – control group (*N* = 118): results of the latent structural equation model of self-regulation, work engagement and emotional exhaustion. All coefficients are unstandardized (standardized coefficients in parentheses). Nonsignificant paths are shown as dashed lines. ^+^*p* < 0.10, *p < 0.05, **p < 0.01, ***p < 0.001.

**Figure 3:**
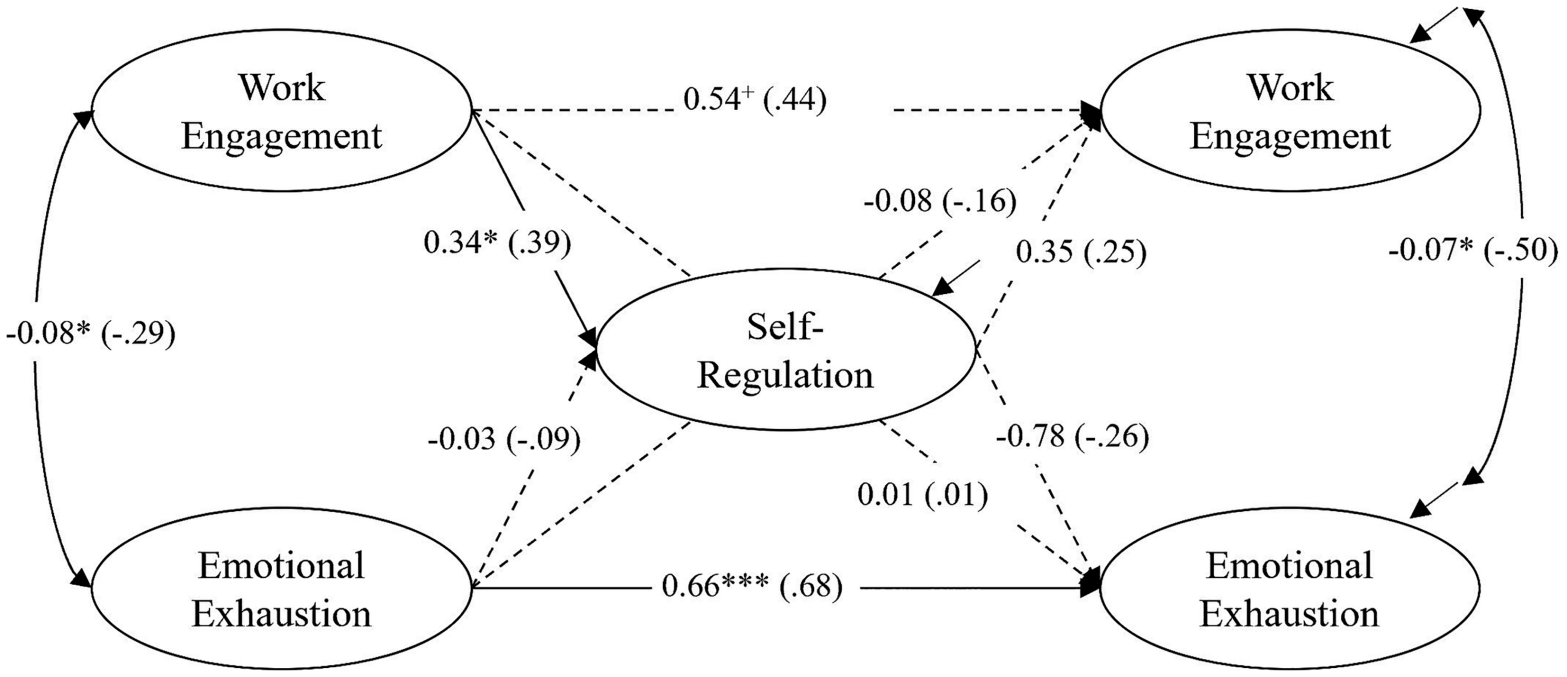
TG – training group (*N* = 95): results of the latent structural equation model of self-regulation, work engagement and emotional exhaustion. All coefficients are unstandardized (standardized coefficients in parentheses). Nonsignificant paths are shown as dashed lines. ^+^*p* < 0.10, *p < 0.05; **p < 0.01; ***p < 0.001.

**Figure 4:**
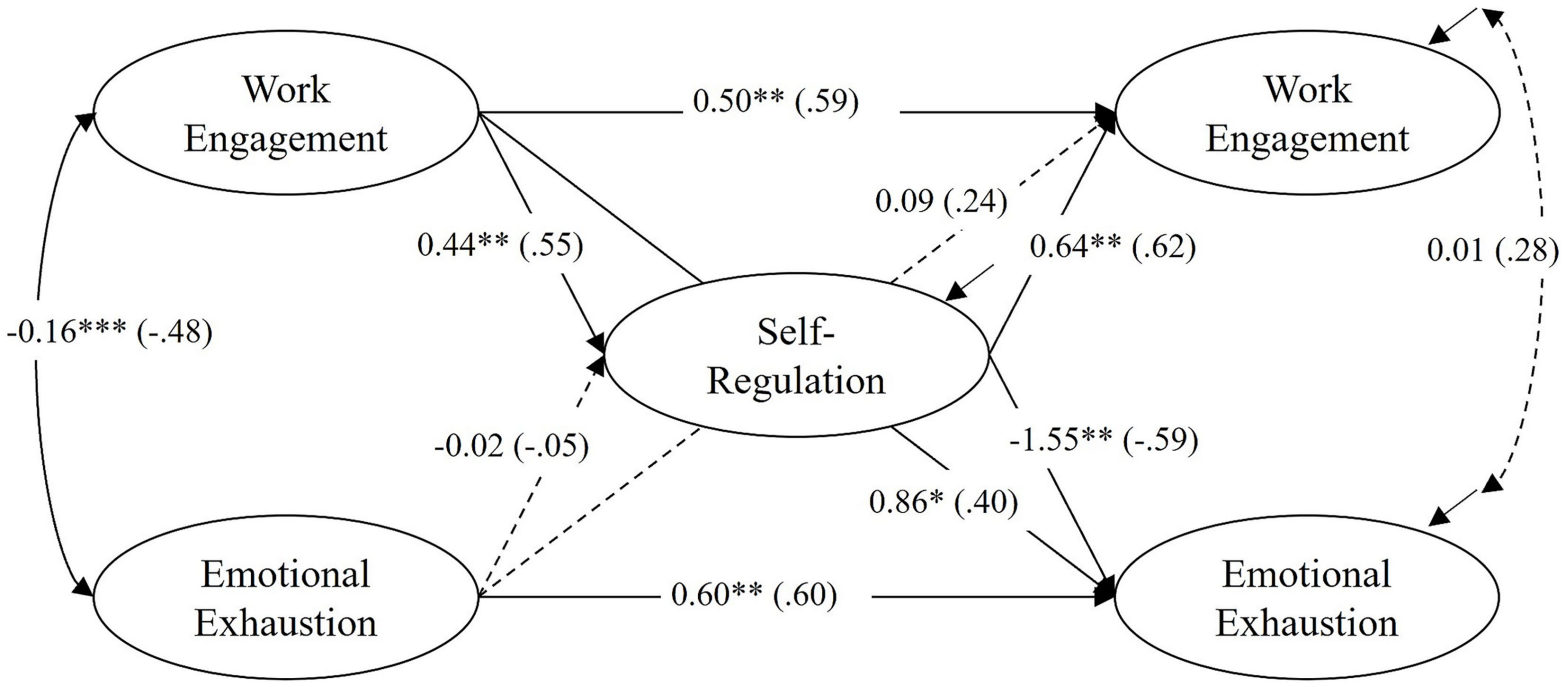
TCG – training plus coaching group (*N* = 60): results of the latent structural equation model of self-regulation, work engagement and emotional exhaustion. All coefficients are unstandardized (standardized coefficients in parentheses). Nonsignificant paths are shown as dashed lines. ^+^*p* < 0.10; *p < 0.05; **p < 0.01; ***p < 0.001.

The model with all tested direct effects (unstandardized and standardized coefficients, *β*) is shown in Figures 2–4 for each group individually. The model fit for the full model was acceptable: χ^2^ = 370.076, df = 270, *p* < 0.000, (corr. MLR = 0.970), CFI = 0.939, RMSEA (90% CI) = 0.064 (0.047, 0.079), Cfit = 0.087. Below, the longitudinal associations of the constructs are described and compared across groups, first considering autoregressive effects, then within-time correlations, and finally cross-lagged effects. The CG model (Figure 2) shows the interaction of the constructs without treatment. Figures 3, 4 show the models for the TG and the TCG, for which treatment took place between T0 and T1 (training for both groups), and between T0 and T2 (coaching for the TCG).

#### 3.2.1 Autoregressive effects

Overall, there were positive, strong to very strong autoregressive effects for all groups. All effects were statistically significant, except for the relationship between work engagement T0-T2 in the TG (*β* = 0.44, *p* = 0.06). The analyses showed a high temporal (inter-individual) stability of work engagement and emotional exhaustion over a period of around seven months. A comparison between the groups showed significant differences in the strength of the effects between the CG and the TCG for work engagement [CG: *β* = 0.86, *p* < 0.001, TCG: *β* = 0.59, *p* < 0.01, *p(∆χ^2^)* = 0.011] and emotional exhaustion [CG: *β* = 0.82, *p* < 0.001, TCG: *β* = 0.60, *p* < 0.01, *p(∆χ^2^)* = 0.037] and a nearly significant difference between the CG and the TG for emotional exhaustion [TG: *β* = 0.68, *p* < 0.001, *p(∆χ^2^)* = 0.065]. This could indicate variability and changes in individual rankings for both constructs in the TCG, and to a lesser extent in the TG.

#### 3.2.2 Within-time correlations

The within-time correlations between work engagement and emotional exhaustion at T0 were all negative, significant, and at a high level (CG: *β* = −0.41, *p* < 0.01; TG: *β* = −0.29, *p* < 0.05; TCG: *β* = −0.48, *p <* 0.001). At T2, however, the correlation was only significant in the TG (CG: *β* = −0.44, *p* = 0.11; TG: *β* = −0.50, *p* < 0.05; TCG: *β* = −0.28, *p =* 0.82).

#### 3.2.3 Cross-lagged effects and results to our research questions

Turning to our first research question, we examined how teachers’ self-regulation, work engagement, and emotional exhaustion interact over time. In our CG model (Figure 2), which illustrates the relationships in the absence of treatment, the hypothesized cross-lagged paths were unexpectedly not significant. Self-regulation at T1 showed only a tendency to reduce emotional exhaustion at T2 (*β* = −0.27, *p* = 0.053; *hypothesis 1*). Regarding the relation between self-regulation and work engagement, a significant cross-lagged effect did appear, but in the opposite direction to what was expected in *hypothesis 2*, namely that work engagement at T0 was a strong predictor of self-regulation at T1 (*β* = 0.60, p < 0.001). Beyond that, no other cross-lagged effects were significant.

Regarding the second research question, we analyzed whether – and to what extent – a self-management training program (TG) or training plus subsequent coaching (TCG) would influence the reciprocal relations between the variables. A comparison of the TG model (Figure 3) with the CG model revealed no significant differences in the relationships between the constructs that can be attributed solely to training (*hypothesis 3a*). Furthermore, the only cross-lagged effect was again the effect of work engagement on self-regulation, but it did not differ significantly in strength compared to the CG [*β* = 0.39, *p* < 0.05; *p(∆χ^2^)* = 0.251]. The effect from self-regulation at T1 on emotional exhaustion at T2 was not significant in the TG (*β* = −0.26, *p* = 0.102), similar to the CG (*hypothesis 1*).

The TCG model (Figure 4), on the other hand, showed several significant differences compared to the CG model (*hypothesis 3b*). There was a strong significant positive effect of self-regulation at T1 on the change in work engagement from T0 to T2 (*β* = 0.62, *p* < 0.01), as hypothesized generally (*hypothesis 2*). This difference was found to be significant compared to the CG [*p(∆χ^2^)* < 0.05]. The comparison with the TG was not significant [*p(∆χ^2^)* = 0.422]. In addition, there was a strong significant negative effect of self-regulation on the change in emotional exhaustion at T2 (*β* = −0.59, *p* < 0.01). This path did not prove to be significantly different from the CG [*p(∆χ^2^)* = 0.222] or the TG [*p(∆χ^2^)* = 0.263]. This is to be expected given the almost significant effect in the other groups.

In addition, there were two significant indirect effects in the TCG: firstly, from work engagement at T0 to change in emotional exhaustion from T0 to T2 mediated by self-regulation at T1, *β* = −0.321 [95% CI (0.086, 1.021)]; and secondly, from work engagement at T0 to the change in work engagement at T2 mediated by self-regulation at T1, *β* = 0.338 [95% CI (−1.121, −0.050)]. These effects were not seen in the other groups.

Furthermore, there was a strong significant positive effect of work engagement at T0 on the change in emotional exhaustion from T0 to T2 (*β* = 0.40, *p* < 0.05). However, the comparison with the other groups was not significant [CG: *p(∆χ^2^)* = 0.547; TG: *p(∆Χ^2^)* = 0.081].

## 4 Discussion

The study examined the interplay between early career teachers’ self-regulation and their well-being, in terms of work engagement and emotional exhaustion over time. In addition, it examined the effects of a self-management training program (SMT) with subsequent online coaching on these interrelations. We applied multi-group structural equation modeling using a cross-lagged panel design and analyzed the reciprocal relationships in three groups – control (CG), training (TG), training plus coaching (TCG) – over a seven-month period.

### 4.1 Key contributions

The *first research question* addressed the general interplay between self-regulation, work engagement and emotional exhaustion. Here, the findings in the CG did not meet expectations. The two hypotheses, according to which higher self-regulation as a personal resource leads to lower emotional exhaustion (*hypothesis 1*) and higher work engagement (*hypothesis 2*) among teachers, were not confirmed in general, or only partly supported. Although we see medium to strong within-time correlations between all three constructs, there were no significant cross-lagged effects in the longitudinal SEM, with one exception: work engagement predicted subsequent self-regulation among teachers. In other words, the more engaged teachers perceived themselves to be, the higher they rated their self-regulation at the following measurement point. This association was observed in all groups and is, therefore, independent of the intervention. This positive effect of work engagement on self-regulation has not yet been described in the literature. We interpret it as an expression of a motivational process: high work engagement – measured using the vitality and absorption at work subscales – supports teachers’ readiness to work in a determined and focused manner and to overcome obstacles at work by persistently persevering even in the face of setbacks and motivating themselves. In the context of the JD-R model, we understand the effect in terms of job crafting, i.e., the proactive shaping of one’s own work resources and work demands (Bakker and Demerouti, 2017). The JD-R model states that individuals with a high level of work engagement are more likely to actively shape their work and thus positively influence their personal and professional resources (Bakker and Demerouti, 2017). We consider cognitive self-regulation in terms of planning, monitoring and evaluation to be an expression of such proactive design of one’s own work processes. This finding underscores the importance of JD-R’s motivation path for teachers’ well-being and shows that cognitive self-regulation can be an expression of actively shaping one’s own work environment and work processes.

Furthermore, the present results only partially support previous findings that self-regulation acts as a protective factor for emotional exhaustion (Mattern and Bauer, 2014). The presumed effect was strong but just short of being statistically significant in the CG model, but it also did not differ from the other groups either. Taking into account the standardized regression coefficients (*β*) greater than 0.2 in all groups, which Orth et al. (2024) consider to be a large effect, it can be assumed that the effect of self-regulation on emotional exhaustion is relevant in all groups. Therefore, *hypothesis 1* can be cautiously confirmed, given the findings of the TCG (see below). However, the results in the CG show that self-regulation does not necessarily impact emotional exhaustion in general. Teachers’ well-being is influenced by a variety of factors, including job characteristics and personal resources, with the influence of the latter being relatively minor (Hascher and Waber, 2021; Mazzetti et al., 2023; Zhou et al., 2024). Previous studies have also found that self-regulation has a rather small effect on teachers’ emotional exhaustion (Mattern and Bauer, 2014). A qualitative analysis of interview data on the coaching process revealed that the participants differed greatly regarding the type of self-regulation goals and goal pursuit processes and, accordingly, benefited to varying degrees in their professional development (Bührer et al., 2024). Against this background, we interpret the lack of a significant relation between self-regulation and emotional exhaustion for the CG and TG as an expression of individual differences in the use and impact of self-regulation skills.

Additionally, the autoregressive paths in the CG model indicate a high stability of work engagement and emotional exhaustion over the course of one semester. These findings are consistent with evidence on the relative stability of psychological and behavioral constructs such as work engagement and burnout, which has been reported in other studies using the JD-R framework (e.g., Hakanen et al., 2008; Vogt et al., 2016), and which is commonly found in autoregressive models (Adachi and Willoughby, 2015). However, this high level of stability is not found in the TCG, suggesting that the SMT and subsequent coaching influenced this stability. This shows that, in addition to a stable core, there are also malleable parts of emotional exhaustion that vary depending on changes in the environment, adaptation processes or interventions (Carstensen et al., 2024).

To address the *second research question*, we examined the impact of the SMT with and without subsequent coaching on the relationships between self-regulation, work engagement and emotional exhaustion. Training alone (TG) does not result in a significant change in the relationship between self-regulation and well-being, instead the combination of SMT and coaching appears to be crucial. The group with SMT and subsequent coaching (TCG) showed significant differences compared to the CG (and partly to the TG) in the type and strength of the relationships between self-regulation, emotional exhaustion and work engagement. *Hypothesis 3a* must therefore be rejected, while *3b* can be accepted. In the TCG, all expected relations proved to be significant, so that even *hypotheses 1 and 2* can be accepted for this group. Self-regulation was directly and indirectly related to emotional exhaustion and work engagement. High self-regulation competencies buffered teachers’ perceived exhaustion (e.g., Mattern and Bauer, 2014) and simultaneously increased their engagement in the workplace (e.g., Bermejo-Toro et al., 2016; De Stasio et al., 2019) – if they participated in the SMT and received coaching while implementing the goals they set in module three. Furthermore, self-regulation acted also as a mediator, showing that work engagement and self-regulation positively influence each other: The more engaged teachers were, the higher their self-regulation, which in turn influenced engagement. These gain cycles could also yield further positive outcomes, for example with regard to the quality of teaching (Klusmann et al., 2008), student outcomes or teacher-student relationships (Dreer, 2023). Moreover, there is evidence that teachers’ self-regulation competencies – as well as their motivation and self-efficacy in promoting these skills – are linked to the development of self-regulation competences in their students (Jud et al., 2024; Karlen et al., 2024). Additionally, higher engagement and stronger self-regulation were associated with a reduction of emotional exhaustion over time. Hence, our results support the assumption that self-regulation can be considered a personal resource for teacher well-being, albeit only when self-regulation is activated as a resource and supported by coaching. It seems to require guided reflection and active engagement with the process of self-regulation during goal implementation, ensuring the maintenance of goal pursuit and the adaptation of volitional strategies (Barato and Rodríguez Moneo, 2022; Jones et al., 2016; Kotte et al., 2018). The fact that the models differed significantly between groups and that strong cross-lagged and indirect effects were found only in the TCG is particularly noteworthy, given that this was the smallest group. In the following section, tentative interpretations for the observed differences and the role of coaching are discussed.

In line with theory and research on cognitive self-regulation of teachers, we consider the structure of the SMT and the *guided goal implementation approach* to be important for effectively enhancing self-regulation competencies and thus making them utilizable as a resource for well-being. The three modules combined knowledge about self-regulation and strategies for behavioral change as well as coaching elements with a goal-implementation approach (Oettingen, 2015). We regard this structured goal-setting approach as central to making the training individually meaningful and to supporting transfer even when obstacles arise. Previous research has shown that a goal implementation process involving mental contrasting and implementation intentions facilitates goal attainment (Oettingen and Gollwitzer, 2001; Oettingen, 2015) by transforming motivation into concrete action. These goal processes, combined with feedback through coaching, prompted teachers to confront and engage deeply with their own self-regulation, occupational situation, and well-being – leading to the observed group differences in the structural relationship between the constructs. The finding that there are no significant differences for the group with only SMT could suggest that, although the SMT initiated a process of goal setting and commitment, the transfer may have been less sustainable. There may have been greater differences within the TG in terms of goal pursuit than in the TCG. While some teachers were able to implement the goals and make behavioral changes in a self-directed manner, others may have found this difficult. Results from the qualitative sub-study of this research project show that coaching plays an important role in all phases of the goal process, especially for persistence and reflection. In addition, different patterns of goal pursuit were identified, which indicates that adaptive support through coaching is essential (Bührer et al., 2024).

With reference to the research literature on coaching, we conclude from the results of our study that coaching is crucial *for transfer* after training and thus persistence in goal pursuit. Various authors point out that the success of PD programs for teachers can be enhanced through individualized and adaptive elements, which effectively support motivation and thus transfer into practice (Lipowsky, 2014; Opfer and Pedder, 2010; Osman and Warner, 2020). In this sense, we assume that, beyond identifying a development goal based on their personal situation, the coaching offered teachers the individualized and flexible support – as well as co-regulation – needed during the transfer and goal-setting process. Coaching represents a personalized learning format that, unlike standardized training, is tailored to individual needs and emphasizes self-directed learning. It supports the implementation of development goals in everyday work and is therefore likely to be more effective than training (Kotte et al., 2018). Coaching may have helped teachers persist in this process, adjust goals where necessary, practice and flexibly adapt strategies, and engage in reflection (Bührer et al., 2024; Grant, 2014). Coaching aims to identify, develop, optimize and utilize resources to support teachers in striving toward behavioral change (van Zyl et al., 2020). In addition, professional support not only strengthens individuals’ persistence but also goal disengagement, which describes individuals’ attempts to distance themselves from a personal goal (e.g., futile goal) to contribute to their well-being and free up for resources to pursue alternative goals (Brandstätter and Bernecker, 2022). We assume that the coaches in our study were able to establish a strong working-alliance and initiate suitable interventions, for example open, systemic and goal oriented questions, and the application of a variety of coaching methods matching the phase of the process (Jones et al., 2016). The coaches professionalism may therefore have contributed to the effectiveness and sustainability of the coaching process. An important mechanism of coaching could be to enable and support the utilization of self-regulation as a personal resource, thereby reinforcing its impact on well-being. This is in line with findings from Barato and Rodríguez Moneo (2022), who stress that coaching is well-suited to strengthening self-regulation competencies, as both share key elements and address similar processes. They show how coaching supports every phase of the cyclical self-regulation process, with particular focus on analyzing the coachee’s situation, setting and defining goals, monitoring actions and guiding the “exploration and management of beliefs, emotions and motivation” (Barato and Rodríguez Moneo, 2022, 10). The latter aspect in particular may have contributed to increased awareness of self-regulation processes and commitment to goals.

We assume that participants in the TCG developed *heightened awareness of the interconnections* between self-regulation, work-related stress factors, and well-being. Other authors (e.g., Maag Merki, 2014) have reported similar conclusions, suggesting that participation in the intervention study probably increased participants’ awareness of or sensitivity to the topic. In our case, the intensive examination of the topic in the TCG may have led to a changed perception of participants’ own abilities, strengths and engagement (e.g., through tools for actively shaping their scope for action and problem solving), as well as of possible challenges (e.g., through self-assessment of work-related behavior). In addition, goal implementation confronted teachers with concrete practice situations requiring them to apply their self-regulation skills. Coupled with the guidance provided by coaching through phases of persistence and disengagement, this likely led to a shift in how stressful situations were perceived and handled, as well as renewed engagement in the classroom.

### 4.2 Strengths and limitations

The strengths of this research lie in its experimental field design, which combined a self-management training program with professional coaching; its incorporation of both positive and negative indicators of well-being; and its use of longitudinal data from in-service teachers over several months. The use of multigroup SEM analyses enabled us to identify reciprocal relationships between self-regulation, emotional exhaustion and work engagement, as well as to demonstrate the impact of a two-phase training program – on-site SMT and online coaching supporting transfer into practice – on these relationships. Significant differences in the structural relationships between latent constructs across groups were observed. To the best of our knowledge, such findings and methodological approaches have rarely been reported in previous research.

At the same time, this study has some limitations that should be considered when interpreting the results. The sample was drawn from primary school teachers who voluntarily participated in a well-established and highly recommended – though not compulsory – PD program at the end of their induction phase. As such, our sample is limited in representativeness. Additionally participants were likely already motivated to pursue further training opportunities and to develop professionally. Furthermore, there is a possibility that participants in all groups showed increased work engagement, and those in the TCG increased goal pursuit, simply because of their participation in the study (Hawthorne effect). Despite the randomized allocation of participants to control and treatment groups, it is nevertheless possible that there was a slight selection bias due to the dropout of less motivated participants – especially in the TCG – as this group was required to make an additional commitment beyond the training (Grant, 2012; Greif et al., 2022). Additionally, the coaching took place online, which could have been perceived as less binding, as described by Greif et al. (2022).

Another limitation lies in the small group size, which limits statistical power. Nevertheless, the effects of self-regulation on work engagement and emotional exhaustion, which proved to be significant despite methodological limitations in the TCG, underscore the importance of training and coaching. However, due to the small sample size, a complete cross-lagged panel model could not be calculated, nor could additional control variables be included. It would therefore be desirable to replicate these longitudinal relations and group comparisons with a larger sample. Future models should also incorporate occupational context factors (e.g., school setting) and personality factors (e.g., learning patterns, personality), as these are likely to have a significant influence on all three variables (Vermunt and Donche, 2017; Skaalvik and Skaalvik, 2011).

Additionally, our results are based only on self-report questionnaires, which could have resulted in common method bias (Podsakoff et al., 2003). However, we opted for self-reports because we were interested in individuals’ perspectives and experiences. Furthermore, even more “objective” measurement methods carry also the risk of bias and are not suitable for measuring subjective experiences of exhaustion, coping with challenges, and engagement. Finally, the cross-lagged panel approach allowed for the prediction of inter-individual differences in changes in emotional exhaustion and work engagement over time, thereby contributing to the understanding of longitudinal relations among the variables. However, this approach does not capture within-person development in the measured variables (Selig and Little, 2012). As such, the results presented here serve as a starting point for further research, such as a more in-depth examination of within-person processes and developments as well as research on the precise mechanisms of coaching.

## 5 Conclusion

The aim of the study was to investigate the longitudinal interplay between the self-regulation of early career teachers and their work engagement and emotional exhaustion, as well as the effects of a self-management training programme followed by online coaching on these interactions. Our results demonstrate an unexpected causal relationship between work engagement and self-regulation across all groups. This points to the importance of motivation and enjoyment of work. We consider the support of these emotional and motivational aspects to be significant for promoting resilience in the teaching profession. The expected direct and indirect effects of self-regulation on work engagement and emotional exhaustion were only evident in the group that received both self-management training and coaching. This study thus provides evidence that self-regulation is a personal resource that can contribute to teachers’ well-being – provided it is activated and supported through coaching. Coaching appears to initiate change processes and thus is a key factor in enabling the use of self-regulation to support teacher well-being. Since both the first years of teaching and the teacher training itself are known to be challenging, we suggest incorporating self-management training with coaching into teacher training programs (Atad and Grant, 2021) and during transitional phases (Carstensen et al., 2024), to reduce emotional exhaustion. Promoting self-regulation may contribute significantly to long-term job satisfaction and retention in the profession. In particular, the positive effect of self-regulation as a personal resource on work engagement is especially relevant for practice, as it may stimulate gain cycles in line with the JD-R model. Given current trends toward individualization and self-regulated learning in education, the training-plus-coaching approach outlined here offers a promising strategy for developing teachers’ own self-regulation skills – as well as for supporting the motivation and engagement needed to promote these skills in the classroom setting.

Our findings reinforce the need for interventions that support teachers’ self-regulation in an adaptive, personalized way. At the same time, it is important to recognize that the standardized self-management training already provided the impetus for active engagement with self-regulation and work-related behavior, laying the foundation for successful goal pursuit. However, training – and coaching in particular – is likely to be effective only when participants are intrinsically motivated and actively engaged in the process.

## Data Availability

The raw data supporting the conclusions of this article will be made available by the authors, without undue reservation.
