# Supplementary material for: Why coaching matters: exploring the interplay of teacher self-regulation and well-being with a longitudinal multigroup model
Source: Front Psychol. 2025 Sep 26;16:1647838. doi: 10.3389/fpsyg.2025.1647838 (PMC12510940; doi:10.3389/fpsyg.2025.1647838)
Supplement: Supplementary file 1 [file Data_Sheet_1.pdf]

Supplementary Material

Table A. Questionnaire Self-Regulation (adapted from Mattern and Bauer, 2014)

SMT Questionnaire: *Bitte geben Sie an, inwieweit die folgenden Aussagen auf Ihr Arbeitsverhalten zutreffen.*	***English translation*** *Please indicate to what extent the following statements apply to your work behavior.*
a) Bevor ich eine umfangreiche Arbeit beginne, lege ich fest, wie ich vorgehe. MB	a) Before I start a big project, I decide how I'm going to do it. MB
b) Bevor ich eine neue Sache in Angriff nehme, mache ich mir meist einen Plan. MB	b) Before I start something new, I usually make a plan. MB
c) Bei einer schwierigen Tätigkeit kann ich gezielt auf die positiven Seiten schauen. (MB)	c) When I'm doing something hard, I can focus on the good parts. (MB)
d) Wenn eine Sache langweilig wird, weiss ich meist, wie ich wieder Spass daran finden kann. (MB)	d) When something gets boring, I usually know how to make it fun again. (MB)
e) Ich kann meine Stimmung so verändern, dass mir dann alles leichter von der Hand geht. (M+B)	e) I can change my mood so that everything becomes easier for me. (M+B)
f) Wenn ich von einer Sache abgelenkt werde, komme ich schnell wieder zum Thema zurück.	f) When I am distracted from something, I can quickly return to the task at hand.
g) Ich kann es verhindern, dass meine Gedanken ständig von der Aufgabe abschweifen.	g) I can prevent my thoughts from constantly wandering away from the task.
h) Nach einer Unterbrechung finde ich problemlos zu einer konzentrierten Arbeitsweise zurück. (MB)	h) After an interruption, I can easily return to a focused way of working. (MB)

Note. Response scale from 1 (disagree) to 4 (agree)

Composition and sources of the items and subscales:

- Action planning: a, b (Kuhl and Fuhrmann, 1998)
- Self-motivation: c, d, e (Kuhl and Fuhrmann, 1998; Mattern and Bauer, 2014)
- Attention control: f, g, h (Schwarzer, 1999)

Table B. Questionnaire Work Engagement (UWES-9, Schaufeli, Bakker and Salanova, 2006)

SMT Questionnaire: *Inwieweit treffen die folgenden Aussagen auf Sie zu?*	***English translation*** *To what extent do the following statements apply to you?*
a) Bei meiner Arbeit bin ich voll überschäumender Energie.	a) At my work, I feel bursting with energy.
b) Beim Arbeiten fühle ich mich fit und tatkräftig.	b) At my job, I feel strong and vigorous.
c) Wenn ich morgens aufstehe, freue ich mich auf meine Arbeit.	c) When I get up in the morning, I feel like going to work.
d) Meine Arbeit reisst mich mit.	d) I get carried away when I'm working.
e) Ich fühle mich glücklich, wenn ich intensiv arbeite.	e) I feel happy when I am working intensely.
f) Ich gehe völlig in meiner Arbeit auf.	f) I am immersed in my work.

Note. Response scale from 1 (does not apply) to 4 (applies)

Table C. Questionnaire Emotional Exhaustion (Baumert et al., 2008; Enzmann and Kleiber, 1989)

SMT Questionnaire: *Inwieweit treffen die folgenden Aussagen auf Sie zu?*	***English translation*** *To what extent do the following statements apply to you?*
a) Ich fühle mich in der Schule oft erschöpft. drin	a) At school, I often feel exhausted.
b) Ich fühle mich insgesamt überlastet. drin	b) I feel overwhelmed overall.
c) Für andere Menschen verantwortlich zu sein, belastet mich sehr.	c) Being responsible for other people puts a lot of pressure on me.
d) Der Zeitdruck, unter dem ich arbeite, ist zu gross.	d) The time pressure I work under is too great.
e) In meinem Beruf wird man ständig überfordert.	e) In my job, you are constantly overwhelmed.
f) Ich habe selten das Gefühl, einmal richtig abschalten zu können.	f) I rarely feel like I can really switch off.
g) Ich fühle mich häufig überfordert.	g) I often feel overwhelmed.

Note. Response scale item a and b: from 1 (does not apply) to 4 (applies); Response scale items c – g 1 (does not apply) to 5 (applies)

Sources of the items:

- a, b (German Version of Maslach Burnout Inventory, Baumert et al., 2008)
- c – g (Enzmann and Kleiber, 1989)
